# Stereotactic body radiation therapy in the re-irradiation situation – a review

**DOI:** 10.1186/1748-717X-8-7

**Published:** 2013-01-05

**Authors:** Frederick Mantel, Michael Flentje, Matthias Guckenberger

**Affiliations:** 1 Department of Radiation Oncology, University of Wuerzburg, Wuerzburg, Germany

**Keywords:** Stereotactic body radiotherapy, Radiosurgery, Re-irradiation, Locoregional recurrence, Normal tissue tolerance, Spinal metastases, NSCLC, Head and neck cancer, Pelvic tumors

## Abstract

Although locoregional relapse is frequent after definitive radiotherapy (RT) or multimodal treatments, re-irradiation is only performed in few patients even in palliative settings like e.g. vertebral metastasis. This is most due to concern about potentially severe complications, especially when large volumes are exposed to re-irradiation. With technological advancements in treatment planning the interest in re-irradiation as a local treatment approach has been reinforced. Recently, several studies reported re-irradiation for spinal metastases using SBRT with promising local and symptom control rates and simultaneously low rates of toxicity. These early data consistently indicate that SBRT is a safe and effective treatment modality in this clinical situation, where other treatment alternatives are rare. Similarly, good results have been shown for SBRT in the re-irradiation of head and neck tumors. Despite severe late adverse effects were reported in several studies, especially after single fraction doses >10 Gy, they appear less frequently compared to conventional radiotherapy. Few studies with small patient numbers have been published on SBRT re-irradiation for non-small cell lung cancer (NSCLC). Overall survival (OS) is limited by systemic progression and seems to depend particularly on patient selection. SBRT re-irradiation after primary SBRT should not be practiced in centrally located tumors due to high risk of severe toxicity. Only limited data is available for SBRT re-irradiation of pelvic tumors: feasibility and acceptable toxicity has been described, suggesting SBRT as a complementary treatment modality for local symptom control.

## Introduction

Despite technological and biological advances in malignant disease management, loco-regional failure remains a frequent challenge after primary radio-(chemo)therapy as recent studies have shown for different entities [1-5]. The locoregional failure rate is 41.7% at three years after concomitant radiochemotherapy for locally advanced head and neck squamous-cell carcinoma (HNSCC) [2]. Locoregional recurrences are reported in up to 85% of the patients after radiochemotherapy for locally advanced non-small cell lung cancer (NSCLC) [6,7]. The locoregional recurrence rate in women treated with radiochemotherapy for cervical cancer was 35% in a met-analysis of 13 trials over all stages (Ia – IVa) [8].

The intention of treatment for locoregional recurrences is manifold and depends on factors like disease type, extent of initial disease and recurrence, metastatic status, primary treatment, interval to locoregional recurrence, performance status, age of the patients and symptoms of the recurrent disease. Consequently, the goal of re-treatment ranges between palliative symptom control, prevention of symptoms due to progressive disease and curative treatment in the absence of metastatic spread.

Locoregional recurrences are a major challenge because therapeutic options are limited in many tumor sites after both primary surgical and radiotherapy treatment. The overall frequency of re-irradiation for locoregional recurrences is unknown. Even in a palliative setting of spinal metastases, re-irradiation is practiced in few patients only [9]. One important reason might be the fear or risk of normal tissue toxicity in the re-irradiation situation.

### Normal tissue tolerance in the re-irradiation situation

Whereas useful guidelines do exist for normal tissue tolerance in the primary situation [10] information in the re-irradiation situation seem to be limited as only few systematic clinical analyses have been performed. Animal models have been used for many decades to evaluate normal tissue tolerance in the re-irradiation situation. While providing the opportunities of systematic study protocols, the transfer of the results from such animal studies into clinical practice must be performed with caution [11]: The simulation of previous oncological and non-oncological therapies or co-morbidities is not possible in animal studies. Due to multimodal therapies many patients e.g. receive chemotherapy before, during or after radiotherapy or are referred to a surgical resection. Combination of different treatment modalities can lead to increased toxicity. Also long-term follow-up is difficult in animal models. Less severe or more subtle toxicity cannot be evaluated in animal studies but might be of clinical relevance. Uniform dose distributions, which are most frequently used in animal studies, are not representative for modern intensity-modulated radiation therapy (IMRT) / SBRT. Lastly, small sample numbers lead to wide confidence intervals especially at the low-risk part of the normal tissue complication probability (NTCP) curve.

Regarding re-irradiation tolerance of acute responding tissue, clinical studies have shown an almost complete recovery within a few months. After full dose re-irradiation in 169 patients with unresectable head and neck cancer, De Crevoisier et al. showed that the incidence of grade 3 mucositis and dermatitis was not significantly different during the first and the second radiotherapy course [12]. In a hyper-fractionated re-irradiation series of 13 patients with recurrent or metastatic abdominal malignancies, only one patient developed grade 3 acute toxicity (abdominal pain and gastro-intestinal bleeding) requiring hospitalization [13].

With regard to re-irradiation of late responding tissue, tolerance is depending on the specific organ at risk. For epithelial and mesenchymal tissues, animal studies suggest almost complete recovery of dermal structures [14]. However clinical data show increased rates of severe toxicity and some studies have shown a dose and volume effect [15,16]. No valid normal tissue complication probability model (NTCP) has been found. Another late-responding tissue complication is osteonecrosis. No clear dose and volume effect has been reported. In an analysis of 115 reirradiated head and neck cancer patients, Salama et al. reported a 11% osteoradionecrosis rate of the mandible after a median lifetime radiation dose of 135 Gy [17]; patients in this study had been treated with a three-dimensional conformal radiation therapy (3D-CRT). Later studies using IMRT showed that sparing of the mandible seems to reduce the rate of osteonecrosis [18,19].

Concerning lung toxicity, good recovery was found in animal models for the acute pneumonitis phase. Recovery took place in 1–3 months and was best for treatment doses less than 75% of the tolerance dose [20]. In contrast, recovery is smaller for late pulmonary fibrosis. For clinical practice this implicates, that re-irradiation in the pulmonary region should be practiced primarily in palliative situations with short life expectancy unless sparing of the lungs is possible. No recovery has been observed in animal studies for the heart, bladder or kidney nor are there any systematic clinical analyses available. Sparing of these organs seems to be of importance.

With respect to spinal cord tolerance in the re-irradiation situation, again animal models have been used for systematic examination of spinal cord damage and recovery. Ang et al. reported a re-irradiation series to the spinal cord in 56 rhesus monkeys: only 4 of 45 animals developed myeloparesis after a first course of 44 Gy followed by re-irradiation course with 57.2 Gy after 1 and 2 years or with 66 Gy after 2 and 3 years, respectively. Recovery of the spinal cord was modelled in two ways: an optimistic model described spinal cord recovery by 76%, 85% and 101% of the initial 44 Gy dose after 1, 2 and 3 years. A pessimistic model described the overall recovery equivalent by 61% [21]. The spinal cord tolerance after re-irradiation with a single fraction radiosurgery was examined in 23 female Yucatan minipigs by Medin et al. [22]. After a uniform dose of 30 Gy in 10 fractions to the spinal cord, the minipigs were re-irradiated with single-fraction radiosurgery and the dose was positioned laterally to the spinal cord resulting in an inhomogeneous dose distribution to the spinal cord (effective dose 50 (ED50) of 19.7 Gy). Identical spinal cord tolerance for radiosurgery was observed whether or not a prior conventionally fractionated radiotherapy had been performed, which indicates full recovery of 30 Gy after 1 year.

Clinical data support the hypothesis of relevant spinal recovery. Analysing the clinical data of 40 patients retreated with conventional fractionation published in 8 different series, Nieder et al. came to the conclusion that the risk of myelopathy is low if cumulative doses to the spinal cord do not exceed 70-75 Gy (2 Gy-fractionated equivalent dose, α/β = 2 (EQD2/2)), single course doses do not exceed 50 Gy (EQD 2/2) and the interval between the two courses is not shorter than 6 months [23]. In case of re-irradiation to the spinal cord with hypo-fractionated stereotactic body radiotherapy, Sahgal et al. performed a case–control study and reported similar results to Nieder et al. [24]: the risk of RM seems to be low if the initial course does not exceed 50 Gy (EQD 2/2), the SBRT-course does not exceed 25 Gy (EQD 2/2) and the interval between the two courses is not shorter than 5 months.

### Rational for using SBRT in the re-irradiation situation

To limit normal tissue toxicity in the reirradiation situation, the target volume is confined to the recurrent macroscopic tumor in most clinical situations without elective nodal irradiation or coverage of larger areas of potential microscopic spread: imaging technologies like FDG-PET are helpful in differentiation of recurrent tumor and post-surgical or radiation induced fibrosis. Highly conformal treatment planning by the use of IMRT or volumetric-modulated arc therapy (VMAT) reduces incidental exposure of normal tissue especially to high irradiation doses; however, a larger low-dose volume needs to be considered. To further reduce irradiation of normal tissue, tight safety margins around the recurrent tumor are vital and can today be realized by the use of daily image-guidance. Effective immobilization of the patient or active motion management strategies need to be applied to minimize intra-fractional uncertainties. Adaptive re-planning might be required in the situation of clinically relevant tumor regression or other anatomical changes during the treatment course.

In this context, stereotactic body radiotherapy needs to be interpreted as a combination of all above-described technologies aiming at best possible accuracy of the whole radiotherapy workflow. Also the short treatment time due to hypo-fractionated delivery of SBRT is to be considered as re-treatment often takes place in a palliative intention.

### SBRT re-irradiation for spinal metastases

Radiotherapy is an essential treatment modality in the management of symptomatic vertebral metastases either for pain palliation, the prevention or control of neurological symptoms or pathological fractures. By using conventionally fractionated radiation protocols with low irradiation doses of 8 – 40Gy in 1 – 20 fractions, good pain response has been reported in the literature [25-27]. However, the achieved duration of pain control is short with a median of 3–6 months [9]. As overall survival has improved substantially in many cancer sites due to modern systemic approaches, re-treatment with conventionally fractionated RT has been used and achieves pain control in approximately 60% of the patients [28] but is performed in only few patients due to the fear of radiation induced myelopathy.

Therefore, several studies investigating SBRT for spinal metastases in preirradiated areas have been performed in the last decade summarized in Table 1[29-37]. All studies used either IMRT, a robotic stereotactic radiosurgery system or tomotherapy for highly conformal dose distributions and daily image-guidance was performed in all studies. However, substantial variability in the reirradiation doses, fractionation and assumed spinal cord recovery is seen in the literature. The median total dose (TD) of the first radiation course ranged from 30–39.2 Gy and the interval between the first course and the SBRT-retreatment ranged between 11–25 months. Median TD for re-irradiation was 30 Gy in most of the trials given in approximately 5 fractions (median 3–22). The accumulated spinal cord dose ranged between 48–83.4 Gy EQD2, indicating no consensus about spinal cord recovery.

**Table 1:** SBRT reirradiation for spinal metastases

**Clinical trial**	**No. patients/treatments**	**Dose 1st RT course (median)**	**Interval (median months)**	**Reirradiation TD/fraction number (median)**	**Accumulated dose to spinal cord EQD2 (median)**	**Planning**	**Set-up/imaging**	**Follow-up (months)**	**Myelopathy**	**Local/pain control**
***Milker-Zabel et al. 2003***	18/19	38Gy	17.7	39.6Gy	n/s	ss-IMRT	Stereotactic	12.3	0%	94.7%
***Mahan et al. 2005***	8/8	30Gy	n/s	30Gy/15	48Gy	Tomotherapy	Daily MV-CT	15.2	0%	100% at time of fu
***Sahgal et al. 2009***	25/37	36Gy	11	24Gy/3	n/s	Cyberknife	kV tracking	7	0%	70%
***Choi 2010***	42/51	40Gy	19	20Gy/2	76Gy	Cyberknife	kV tracking	7	n=1 G4	73%
***Sterzing et al. 2010***	36/36	36.3Gy	17.5	34.8Gy/11	46.5Gy	Tomotherapy	Daily MV-CT	7.5	0%	1-year 76%
										2-years 63%
***Damast et al. 2010***	95/97	30Gy	n/s	20-30Gy/5	54.3Gy	IMRT	Daily portal images or CBCT	12.1	0%	1-year 66%
***Garg et al. 2011***	59/63	30Gy	n/s	27-30Gy/3-5	n/s	IMRT	Daily CT on rails or CBCT	13	n=2 G3 peripheral nerve injury	76%
***Mahadevan et al. 2011***	60/81	30Gy	20	24-30Gy/3-5	n/s	Cyberknife	kV tracking	12	n=3 persistent radicular pain	93%
									n=1 lower-extremity weakness	
***Chang et al. 2012***	49/54	39.2Gy	25	27Gy/3	83.4Gy	Cyberknife	kV tracking	17.3	0%	79%

Abbreviations: RT = radiotherapy; TD = total dose; n/s = not specified; ss-IMRT = single shot intensity-modulated radiation therapy; MV-CT = megavolt computed tomography; kV = kilo Volt; CBCT = cone beam computed tomography; IMRT = intensity modulated radiation therapy; G = grade.

Overall, a very low incidence of myelopathy was observed. In the study conducted by Choi et al. one patient of 42 (incidence 2%) developed a grade 4 spinal cord neurotoxicity. This patient had received a spinal cord total dose of 40 Gy for metastatic breast cancer in the first course and a stereotactic radiation surgery for a 10.5 cm^3^ T5 recurrence with 20 Gy in 2 fractions. Dose was prescribed to the 80% isodose line. The maximal spinal cord dose in the SBRT course was 19.25 Gy [30].

Garg et al. observed 2 of 59 patients (incidence 3.3%) with grade 3 neurological toxicity related to nerve damage without loss of independent ambulatory function [32]. Mahadevan et al. described 4 patients of 60 (6.7%) with persistent or even worsening neurological symptoms, yet all of them having corresponding radiological progression [33]. There were no cases of myelopathy in the other studies.

Local control (LC) ranged from 63% to 100% and therefore seems to be very promising (details see Table 1). Damast et al. analysed the largest study population: 97 tumor sites were retreated with image-guided IMRT, resulting in a local control rate of 66% at one year. Patients in this study were either re-irradiated with 5 × 4 Gy or 5 × 6 Gy. The comparison between the two dose groups showed a significant difference in cumulative local failure after one year: 26% for the 30 Gy group and 45% for the 20 Gy group [31]. Milker-Zabel et al. reported a local control rate of 94.7% after a median follow-up of 12.3 months in 18 patients (19 lesions) re-treated with either fractionated stereotactic conformal RT or IMRT [35]. No local recurrences at the time of follow-up were observed in the analyses by Mahan et al., but only eight patients were re-treated in this study of whom one died of distant disease briefly after treatment and one 11 months after treatment [34]. Mahadevan et al. reported SBRT for patients with progressive epidural disease: at a median follow-up time of 12 months they observed a 93% rate of stable or improved epidural disease, results equally to surgery in this situation [33]. Some studies examined pain control as an endpoint: Damast et al. reported a significant, mild or no pain relief in 46%, 31% and 23% of the patients, respectively [31]. Pain relief occurred in 13 of 16 patients (81%) in the analysis of Milker-Zabel et al. [35]. Sterzing et al. reported a reduction of tumor-related pain on a visual analogue scale (0 – 10, 0 = no pain; 10 – worst imaginable pain) from a median value of 7 down to a median value of 3 after 6 weeks (p = 0.0046) [37].

In conclusion, these findings suggest that SBRT is a safe and effective treatment modality in the re-treatment situation when conventional radiotherapy is unable to be within spinal dose tolerance. However, further knowledge about spinal recovery and spinal cord tolerance to inhomogeneous SBRT dose distributions is required.

### SBRT re-irradiation for lung tumors (NSCLC)

In locally advanced lung cancer, locoregional recurrence remains high in up to 85% of patients [6,7]. Intrathoracic relapse in previously irradiated areas of patients is a frequent challenge in clinical routine. However, re-irradiation of NSCLC is performed in only 1.5% to 8.1% of all patients [7,38,39].

In 2011 Jeremic et al. reviewed 11 studies (two of them prospective) of conventionally fractionated external beam radiation therapy (EBRT) re-irradiation for recurrent NSCLC. The analyses showed an improved overall survival (OS) after retreatment with higher doses compared to low-dose retreatments accompanied by increased rates of grade 2–3 pneumonitis and esophagitis [40]. The rational for SBRT therefore is safely escalating the irradiation dose while minimising toxicity (Table 2).

**Table 2:** SBRT reirradiation for lung cancer

**Clinical trial**	***Poltinnikov et al.***	***Coon et al.***	***Kelly et al.***
**No. patients/treatment**	17/17	12/12	36/36
**Dose 1**^**st**^**RT course**	≥ 50 Gy	n/s	median 61.5 Gy
			(range, 30–79.2 Gy)
**Interval (median)**	n/s	n/s	22.0 months (range, 0–92 months)
**Total re-irradiation dose**	median 32Gy (17.5 – 42.0)	60Gy	50 Gy (72%)
			40 Gy (17%)
			Other (11%)
**Single fraction dose**	median 4Gy (2.5 – 4.2)	20Gy	12.5 Gy (72%)
			10Gy (17%)
			Other (11%)
**Technology**	SBF	Cyberknife	SBF, 4D-CT, FDG-PET
**Target size**	median field size 95cm^2^	median GTV 14.3cc	Tumor size (median) 1.7cm
	(30–189)		
			(range, 0.6–3.8 cm)
**Symptom relief**	11/13	n/s	n/s
**Median follow-up (range)**	n/s	12 months	15 months (4–45)
**Local control**	5/17 responders	92% @ 1a	92%
**Median Overall survival (range)**	5.5 months (2.5–30)	67% @ 1a	59% @ 2a
**Toxicity**	G2 esophagitis n=4	No G3 toxicity	At least one G3 in 33% of patients
	G2 pneumonits n=1		
			G3 peunomitis n=7
			G3 esophagitis n=3
			G3 Skin ulcer n=2
			G3 Cough n=1
			No G4/5 toxicities

Abbreviations: RT = radiotherapy; n/s = not specified; SBF = stereotactic body frame; 4D-CT = 4-dimensional computed tomography; FDG-PET = fluorodeoxyglucose positron emission tomography; G = grade; @ = at time of; a = year.

Poltinnikov et al. examined the outcome and dosimetric predictors of acute esophagitis in 17 NSCLC patients receiving re-irradiation using a stereotactic body frame (SBF) [41]. Hypofractionated 3-dimensional RT was used with a median RT dose of 32 Gy (range, 4 – 42 Gy) and a median fraction size of 4 Gy (2.5 – 4.2 Gy). Multiple noncoplanar RT fields (median 6) were used in each patient to minimize spinal cord exposure. In this study re-irradiation was defined as treatment of areas that had received a full RT of 50 Gy or more 13 patients had symptoms as pain or shortness of breath. After re-treatment, 11 of these 13 symptomatic patients had improved or even complete disappearance of symptoms. Grade 1 and 2 acute esophagitis was observed in 7 patients, however symptoms disappeared within 3 months after RT. One patient suffered from pneumonitis grade 2. Notably, no grade 3 toxicities and no bronchial or vascular toxicities were observed. Radiologic response rates before death or at the time of last follow-up showed one patient with complete response, 4 patients with partial response and 5 patients with stable disease whereas 7 patients had disease progression. Median overall survival time was 5.5 months (2.5 – 30 months).

In an analysis of 51 NSCLC patients treated with the Cyberknife Radiosurgery Robotic System Coon et al. evaluated the role of positron-emission tomography in the planning and evaluation of RT treatment response. Among these patients 12 patients with recurrent disease after definitive treatment were described [42]. Re-treatment doses in these patients were three fractions of 20 Gy whereas no information was given about the initial radiation doses or treatment intervals. Treating volumes were substantially smaller compared to the study of Poltinnikov et al. with a median gross tumor volume of 14.3 cm^3^ compared to a median field size of 95 cm^2^, respectively. Local control rates and overall survival rates one year after reirradiation were 92% and 67%, respectively. Similar to the study mentioned above no G3 toxicities occurred.

Kelly et al. retrospectively reviewed the medical records of 36 patients treated with SBRT for relapse of NSCLC who had been previously irradiated to the chest [43]. In-field relapse was defined as a recurrence in an area that had received an initial dose of more than 30 Gy in the first EBRT course. The median tumor size was 1.7 cm for SBRT re-irradiation and most of the patients received 50 Gy in 4 fractions. The median initial EBRT dose was 62 Gy and the median interval between EBRT and SBRT 22 months. The median follow-up time after SBRT was 15 months in this study. The overall 2-year survival was 59% and 2-year progression-free survival was 26%: the most frequent site of failure was intrathoracic. However, only 13.6% (3 patients of 22) of these intrathoracic relapses occurred inside the SBRT field and the authors mention that planning target volume (PTV) dose coverage and/or total dose and fractionation was suboptimal in two of the three patients. At least one form of late grade 3 toxicity was observed in 12 patients (33%). Grade 3 pneumonitis was experienced by 7 patients and with 11 patients suffering from grade 2 pneumonitis the latter was the most frequent toxicity in this analysis. Three patients developed grade 3 esophagitis, two grade 3 chest wall ulcers, and two patients developed grade 3 cough. Overall no grade 4 or 5 toxicity occurred.

In conclusion, few studies with small patient numbers are available about SBRT for re-irradiation of NSCLC. Definition of re-irradiation differs among the studies as well as total doses and fractionation schemes. Overall survival seems to depend particularly on patient selection and is limited by systemic progression. All investigations do show promising local control rates that seem to exceed RT with conventional techniques. Furthermore small field SBRT for re-irradiation to the chest seems to be safe.

SBRT for reirradiation after prior SBRT has been evaluated in a retrospective analysis of 32 patients by Peulen et al. [44]. 29 patients received two SBRT courses, two received three and one patient even four SBRT courses. Re-irradiation was defined as an overlap of more than 50% of the PTV. The median interval between the two SBRT courses was 14 months and the median follow-up time from the beginning of re-irradiation 12 months, respectively. Most patients (n = 23) were treated for pulmonary metastases. The results showed eight patients with grade 3–4 toxicity and three patients suffered from grade 5 toxicity and died of massive haemorrhage. All patients with grade 4–5 toxicity were treated for centrally located tumors. Additionally, larger clinical target volumes (CTV) were associated with severe toxicity and poorer local control. LC 5 months after re-treatment was 52% with actuarial 1-year and 2-year survival rates of 59% and 43%, respectively.

Consequently, a second course of SBRT with overlapping target volumes should only be considered in cases of small recurrent tumors and peripheral location.

### SBRT re-irradiation for head & neck cancer

Therapy options for locoregional recurrences in previously irradiated head and neck patients are limited. Salvage surgery is the standard treatment for small relapses [45,46]. But with further locoregional spread, surgery alone is not sufficient. In the past patients considered inoperable often received palliative chemotherapy with low response rates ranging from 10% to 40% [47,48]. Salama et al. reported the long-term outcome of concurrent treatment with chemotherapy and re-irradiation of patients suffering from recurrent or second primary squamous cell carcinoma of the head and neck (SCCHN) [17]. Full-dose re-irradiation in this multimodal therapy setting showed promising results for long-term survival. 115 patients had been treated with conventional RT techniques for the initial and the second course. Locoregional control and freedom from distant metastasis rate at three years was 51% and 61%, respectively. Re-treatment was highly toxic with 19 treatment related toxic events, five of them due to carotid haemorrhages. The authors came to the conclusion that re-irradiation of recurrent head and neck cancer should be limited to clinical trials.

As normal tissue toxicity was significant after conventional RT, the introduction of SBRT in the re-irradiation of head and neck cancer patients has been pursued in the last years. Several approaches for SBRT have been published recently.

Voynov et al. reviewed 22 patients re-treated with SBRT in recurrent previously irradiated SCCHN [49]. Using the Cyberknife patients were irradiated with a median single fraction dose of 5 Gy for a median of 5 fractions (details see Table 3). Re-treatment with SBRT was considered to be safe as no grade 4 or 5 toxicities or late toxicities occurred. In 2009 Roh et al. published an analysis of 36 patients with recurrent head and neck cancer re-irradiated with Cyberknife radiosurgery (RS) with three fractions of 10 or 13 Gy (34 sites of 44). The authors changed to a fractionation schedule of five fractions of 5 Gy or 8 Gy [50]. 15 patients (42.9%) showed complete tumor regression and 13 (37.1%) partial regression with median local control rates and overall survival rates after one year of 61% and 52.1%, respectively. Late adverse events were observed in three patients: two grade 4 and one grade 5 toxicity: one patient suffered from mandible bone necrosis five months after RS; the treated submandibular tumor mass abutted the mandible in that case. Two patients developed a chronic mucosal ulcer, trismus and skull-base necrosis 4–6 months after treatment. All of these patients had received a full dose radiation of 66 to 70.2 Gy in the first RT-course and a RS dose of 30 to 39 Gy in 3 fractions. The authors mentioned that these RS doses might be too high: 30 to 39 Gy in 3 fractions are equivalent to 80-130 Gy in conventionally 2 Gy-fractionated radiotherapy based on an α/β of 3 Gy. After adapting the dose and fractionation protocol to 25–40 Gy in 5 fractions (biologically equivalent dose 40–90 Gy_2_) no late complications have been observed so far. Besides dose and fractionation the inter-/intratreatment mucosal movement due to swallowing and respiration was mentioned as a risk for chronic mucosal ulceration.

**Table 3:** SBRT reirradiation for head and neck cancer

**Clinical trial**	***Voynov et al. 2006***	***Heron et al. 2009***	***Unger et al. 2010***	***Roh et al. 2009***	***Ozyigit et al. 2009***	***Vargo et al. 2012***
**n**	22	25	65	36	24	34
**Initial therapy dose**	median BED_10_ 97.8 Gy (70.1 – 190.3 Gy)	median, 64.7 Gy	median 67 Gy (32–120 Gy)	median, 70.2 Gy (39.6 – 134.4 Gy)	median, 70 Gy (48–70 Gy)	median 61.2 Gy (42–157 Gy).
**Interval**	n/s	13 months (range, 5–94 months)	26 months (range, 2–318 months)	24 months (range, 3.1–252.6)	38 months (range, 10–242 months)	53 months (range, 1–302 months)
**Re-irradiation dose**	5 (1 – 8) x 5 Gy (3 – 16 Gy)	5 x 5 Gy	5 (2–5) x 6	3 x 10/13 Gy	5 x 6 Gy	median dose of 40 Gy in 5 fractions (interquartile range, 30–44 Gy)
		5 x 6.4 Gy	Gy (4–12Gy)	5 x 5/8 Gy		
		5 x 7.2 Gy				
		5 x 8.0 Gy	median 30 Gy (21–35 Gy)			
		5 x 8.8 Gy				
**Target size (median)**	TV 19.1 cm^3^ (range, 2.5 – 140.3 cm^3^)	TV 44.8 mm^3^ (range, 4.2–216.6 mm^3^)	Target volume 75 cm^3^ (range, 7–276 cm^3^)	GTV 22.6 cm^3^ (range, 0.2 to 114.9 cm^3^)	TV 63.4 cm^3^ (range 26.3–170.4 cm^3^)	TV 19,6ml (range, 4.5 – 103.9 ml)
**Median follow-up time**	19 months (range 11–40 months)	n/s	16 months	17.3 months	24 months	10 months (range, 0–55 months)
**Local control**	26% @ 2 years	n/s	30% @ 2 years *	61% @ 1 year	82% @ 2 years	77% @ 6 months
				52.2% @ 2 years		59% @ 1 year
**Overall survival**	22% @ 2 years	median 6 months (95% CI 5–8 months)	12 months	52.1% @ 1 year	cancer specific survival	76% @ 6 months
			41% @ 2 years *	30.9% @ 2 years		
					64% @ 2 years	59% @ 1 year
**Toxicity**	1/22 grade 2,	3/25 grade 1	19/65 acute grade 1–3 toxicities	13/36 grade 3 acute toxicities	5/24 severe late side effects (grade ≥ 3)	Acute/late grade 3 toxicity was 15/6%, with no grade 4–5 toxicity
	1/22 grade 3 mucositis	1/25 grade 2				
		No grade 3–5 toxicities		3/36 late toxicity: 1 bone necrosis, 2 soft tissue necrosis		
	No grade 4/5 toxicitiy					
			6/65 late grade 4 toxicities: arterial bleeding, soft			
			tissue necrosis, fistula formation			
			1 treatment related death			

Abbreviations: BED_10_ = biologically effective dose (α/β of 10 for acute/tumor effects); n/s = not specified; @ = at time of; TV = tumor volume; GTV = gross tumor volume.

* definitively treated patients.

Unger et al. reported on stereotactic RS reirradiation in 65 head and neck cancer patients [51]. Treatment sites were oropharynx (n = 13), hypopharynx (n = 8), naso-pharynx (n = 7), paranasal sinus (n = 7), neck (n = 7), and others (n = 23). Patients were treated with SBRT reirradiation definitively (n = 38) or in palliative intention due to metastatic or untreated local disease (n = 27). Nine patients had complete macroscopic resection before SBRT and 33 patients were treated with simultaneous chemoradiotherapy. Initial RT dose was median 67 Gy (32–120 Gy) and median treatment interval between initial course and SBRT 26 months. Most patients were treated with 5 x 6 Gy (2–5 × 4–12Gy) but dose and fractionation was individualized by the treating physician and total dose was commonly reduced after complete macroscopic resection. For 56 patients evaluable for response, complete, partial and no response was observed in 30 patients (54%), 15 patients (27%) and 11 patients (20%), respectively. Median overall survival was 12 months. The OS rate was 41% for definitively treated patients and the locoregional control rate 30% in these patients. The authors reported that nasopharynx site, surgical resection and higher total dose were significantly correlated with improved locoregional control and nonsquamous histology and surgical resection with improved OS. One patient died of unspecified causes 2 weeks after reirradiation, considered treatment related. Grade 4 late toxicities were observed in six patients (9%) including soft tissue necrosis, arterial bleeding, fistula formation and severe dysphagia. No association of higher RS dose or higher cumulative dose with severe late complications was seen, though quantitative analysis were limited due to the small number of events.

Heron et al. conducted a phase I dose escalation trial of re-irradiation with SBRT for recurrent SCCHN in 2009 [52]. 25 SCCHN patients were reirradiated either with 5 × 5 Gy (n = 3), 5 × 6.4 Gy (n = 3), 5 × 7.2 Gy (n = 3), 5 × 8 Gy (n = 6) or 5 × 8.8 Gy (n = 10). The authors observed treatment response in 76% of the patients. However, they could not correlate dose and tumor size to the probability of local control. The authors could not find an association between dose and toxicity due to the small number of adverse events. The highest dose applied (44 Gy) was considered to be adequate for a phase II trial. To increase efficacy of SBRT reirradiation, later on Heron et al. investigated combinations of SBRT with cetuximab [53]. Therefore they compared two patient groups in a retrospective-matched cohort study: 35 patients were treated with SBRT reirradiation alone or with additional weekly cetuximab during SBRT, respectively. Toxicities were comparable in both patient cohorts and an overall survival benefit was seen for the cetuximab arm. Feasibility and efficacy of SBRT reirradiation with or without cetuximab also was shown recently by Comet et al. [54].

In a retrospective analysis of 51 patients re-irradiated for locally recurrent nasopharyngeal carcinoma (LRNPC) Ozyigit et al. compared 24 patients treated with SBRT to 27 patients treated with 3D-CRT [55]. Median total dose in the first treatment course was 67.4 Gy (range, 59.4–70 Gy) for the 3D-CRT group and 70 Gy (range, 48–70 Gy) for the SBRT group (p = 0.1). Median total reirradiation dose was 57 Gy with a single fraction dose of 2 Gy per day using 3D-CRT. Patients in the SBRT arm were retreated with a total dose of 30 Gy delivered over 5 consecutive days. There was no significant difference in the 2-year actuarial local control rate (82% and 80% for the SBRT and 3D-CRT group, respectively, p=0.6) and the 2-year actuarial cancer-specific survival (64% and 47% for the SBRT and 3D-CRT group, respectively, p=0.4). In contrast the authors observed a significant difference in the rate of late side effects. Serious late toxicities occurred in 21% of the patients treated with SBRT compared to 48% of the patients treated with 3D-CRT. The follow-up was similar for SBRT and 3D-CRT with median 23 months and 24 months, respectively, and patient characteristics were comparable as well. Taken into account that serious late toxicities after reirradiation of LRNPC had been reported before to be as high as 15-45% [56-59] SBRT therefore seems to be an attractive treatment modality with less late side effects and shorter treatment times for the re-irradiation of LRNPC.

Vargo et al. recently reported on SBRT in recurrent, nonsquamous cell cancers of the head and neck [60]. 34 patients were re-irradiated with a median SBRT dose of 40 Gy in 5 fractions. The toxicity and quality of life were followed prospectively with a median follow-up of 10 months. The authors found promising local control rates of 77% and 59% after 6 months and 12 months, respectively. For tumors < 25 ml they reported a significant improvement of local control compared to larger tumors (> 25 ml). Acute and late grade 3 toxicities occurred in 15% and 6% of the patients respectively and no grade 4 or 5 toxicities were observed.

In conclusion, SBRT in the re-irradiation situation for head and neck tumors is a promising salvage therapy modality with encouraging local control rates and justifiable toxicities. Severe late adverse events (grade 4–5 toxicities) have been reported in some studies but are less frequent than in patients re-treated with conventional techniques. Very high single fraction doses of 10–13 Gy or higher should be avoided. Up to now, no investigations have been performed for comparison of SBRT and IMRT reirradiation. The latter has been analyzed for reirradiation of head and neck cancers over the last years showing encouraging results as well.

### SBRT re-irradiation for pelvic tumors

Primary treatment of pelvic cancers such as adenocarcinoma of the rectum, prostate cancer or gynaecological tumors often includes radiotherapy. The locoregional recurrence rate in women treated with radiochemotherapy for cervical cancer is 35% over all stages [8] and 3% to 15% in patients treated for rectal adenocarcinoma [61]. Patients are frequently not considered eligible for salvage surgery especially in the case of lateral pelvic wall involvement or tumor abutment to the iliac vessels [62].

Dewas et al. presented a retrospective study of 16 patients reirradiated with Cyberknife for lateral pelvic recurrences [63]. Patients were treated for recurrences of primary anal canal cancer (6 patients), rectal cancer (4 patients), uterine cervix cancer (4 patients), endometrial cancer (1 patient) and bladder carcinoma (1 patient). Patients had been treated before with a median RT dose of 45 Gy (range, 20 Gy – 96 Gy), median interval between first RT course and retreatment was 5.1 months. In the second course using SBRT, 36 Gy in 6 fractions were delivered over three weeks. The median follow-up was 10.6 months (1.9 to 20.5 months). The authors reported a 1-year local control rate of 51.4%, median disease-free survival of 8.3 months and actuarial one-year survival of 46%. Adenocarcinoma showed a tendency for better local control compared to sqamous cell carcinoma (p = 0.09). Acute and late toxicities were limited to grade ≤ 2.

Guckenberger et al. [64] reported a series of 19 patients using SBRT for treatment of locally recurrent cervical (n=12) or endometrial (n=7) cancer; three patients were re-irradiated after adjuvant external beam radiotherapy (n=1; conventionally fractionated 50 Gy) or definitive external beam radiotherapy and brachytherapy (n=2; conventionally fractionated 50–56 Gy; brachytherapy 3 × 8 Gy and 5 × 8.5 Gy) in the primary situation. Patients were treated with 3 × 10 Gy (n=2) or 4 × 7 Gy (n=1) with dose prescription to the 65% isodose. No acute toxicity was observed but one patient developed an ileus 6 months after SBRT. All patients were locally controlled but follow-up was short ranging between 8 and 18 months. Two patients died 8 and 23 months after SBRT.

In 2009 Deodato et al. published preliminary results of SBRT in local and distant recurrences of gynaecological tumors (RGT) [65]. The analyses included 11 patients treated with SBRT of whom 6 received SBRT as a reirradiation. Previous RT doses ranged from 37.5 to 65 Gy (median 50.2 Gy). 8 patients had been pretreated with chemotherapy, and 5 patients with surgery. Re-irradiation was performed with 5 × 4 Gy, 5 × 5 y or 5 × 6 Gy on five consecutive days on base of an institutional dose-escalation protocol. PTV ranged from 4.0 cc to 273.0 cc (median, 42.0 cc). No patient showed acute toxicity or late toxicity grade > 2. With a median follow-up of 19 months (range, 2–37 months) the authors reported a 2-year local progression-free survival (LPFS) of 81.8% and a 2-year metastases-free survival of 54.5%. In a subgroup analysis the 2-year LPFS of patients treated with the highest dose of 30 Gy was 87.5% and 66.7% for the remaining population indicating better local control for the higher treatment dose.

Recently, Jereczek-Fossa et al. reported on SBRT for solitary recurrent primary, lymph node, or metastatic prostate cancer [66]. 34 patients (38 lesions) were treated with SBRT using the robotic Cyberknife system: 15 patients were reirradiated for local recurrence, 4 patients were reirradiated for anastomosis recurrence, 16 patients were treated for single lymph node recurrence and 3 patients were treated for single metastasis. Median SBRT dose was 30 Gy in 4.5 fractions and was performed as a reirradiation in 27 of the 38 treated lesions, that is the recurrent lesion was located in the previously irradiated volume. 18 patients received androgen deprivation (median duration, 16.6 months) in addition to SBRT. The median follow up was 16.9 months (range, 3 – 35.2 months). The mean interval between primary diagnosis of prostate cancer and time of SBRT was 66 months (24 – 180 months). 7 acute urinary events (18% of all treatments) were reported (3 cases of grade 1, 2 cases of grade 2, and 2 cases of grade 3 events). Grade 3 acute urinary events included macroscopic hematuria and a worsening of urinary incontinence already existing before SBRT due to prostatectomy. All but one acute urinary event developed in patients with local/anastomosis recurrence. Only one acute rectal grade 1 event occurred (3% of all patients). The authors observed 7 late urinary events (3 cases of grade 1, 2 cases of grade 2 and 2 cases of grade 3 events). Grade 3 late urinary events included transient macroscopic hematuria and a persistent worsening of urinary incontinence, that occurred in the same patient who had an acute grade 3 event. These late urinary toxicities were observed in patients treated for local recurrence to the prostate region and patients treated for lymph node recurrences. Two late rectal events were reported (1 grade 1 and 1 grade 2, not otherwise specified). Biochemical response was reported in 32 of 38 lesions. Considering lesions treated with SBRT alone (no concomitant systemic therapy), a complete PSA response was observed in 9 lesions (56.25%) and a partial PSA response in 4 lesions (25%). Disease progression was reported in 14 lesions after a median of 10 months (range, 2 – 17 months) but in-field progression occurred in only 3 lesions. Time to progression was 11 months when androgen deprivation was added to SBRT and 10 months without. Progression-free survival rate was 42.6% at 30 months. With regard to these data SBRT as retreatment for locally or regionally recurrent prostate cancer seems to be practicable with good in-field tumor control and a low toxicity rate. Further analyses are needed to validate these findings and to identify patients with best benefit of SBRT treatment.

Defoe et al. analyzed 14 patients treated with SBRT for presacral recurrent adenocarcinoma of the rectum [67]. All patients had been previously irradiated with a median of 50.4 Gy (range, 20 – 81 Gy). The treatment dose for reirradiation was 36 Gy in 3 fractions for 11 patients and 3 patients were treated with a single fraction of 12, 16 or 18 Gy. Median tumor volume was 52.5 cc (range, 19 – 110 cc). Time of median follow-up was 16.5 months (range, 6 – 69 months). The one- and two-year LC rates were 90.9% and 68.2%, respectively. Overall survival was 90% after one year and 78.8% after two years. Neither dose and fractionation nor tumor volume were significantly correlated with LC and OS. No grade 3 or 4 genitourinary, gastrointestinal, or neurologic toxicities occurred. One patient suffered from a recurrent pelvic abscess after SBRT, with need for drainage. Seven patients (50%) reported pain with recurrence before retreatment with SBRT and four of these patients (57.1%) had no pain after SBRT without any need for analgetic medication. SBRT in this small patient cohort has been an efficient and well-tolerated treatment for presacral recurrent rectal cancer.

In summary the role of SBRT in the reirradiation of pelvic recurrences is currently poorly investigated. Recurrences from gynaecological tumors are most frequently treated in palliative intention with chemotherapy [68]. In case of lateral pelvic tumor recurrence, retreatment with surgery is rarely possible due to pelvic wall or iliac vessel involvement. Similarly, patients suffering from presacral recurrence of rectal cancer often are not amendable for curative resection, but even without treatment do have a median survival of up to 8 months [69]. If no treatment is offered or even if chemotherapy alone without any local treatment is given, these patients develop local complications particularly pain with dramatic decrease of their quality of life [70]. SBRT therefore might be a well-tolerated and short treatment to palliate local symptoms and achieve improved local control. In recurrent prostate cancer after primary treatment, androgen deprivation is indicated for most patients. As surgery is rarely performed in these patients due to advanced age and tendency for systemic dissemination, SBRT might be a feasible and safe treatment supplement to systemic hormonal therapy.

## Conclusion

Locoregional recurrences are a frequent challenge in oncology. Nevertheless, conventional radiotherapy for re-irradiation has been performed in only few patients due to the risk of normal tissue toxicity. SBRT may allow a paradigm shift in this important clinical setting, where alternative treatment options are limited.

The recurrent macroscopic tumor is precisely visualized using modern multimodal imaging technologies and the target volume is confined to gross tumor volume without inclusion of elective nodal regions or volumes of potential microscopic tumor extension. Safety margins are minimized by accurate, image-guided treatment delivery and highly conformal treatment planning further reduces exposure of normal tissue. SBRT combines these technologies and allows hypo-fractionated dose escalation in these small target volumes. Shorter treatment times of SBRT are a considerable advantage in this clinical setting as re-treatment often takes place in a palliative intention.

This small volume but intensified SBRT re-irradiation has shown promising results with high rates of local tumor and symptom control and simultaneously low rates of severe normal tissue toxicity. Most mature results have been described for SBRT re-irradiation of vertebral metastases with high rates of pain and local control; toxicity, especially radiation induced myelopathy, was low in all series. Re-irradiation in the head-and-neck, thoracic and pelvic region has gained interest with availability of SBRT and preliminary clinical results are promising.

However, most if not all data available about SBRT re-irradiation are based on small single institution studies with limited follow-up. The details of SBRT practice in terms of patient selection criteria, total irradiation dose, fractionation, normal tissue dose constraints and target volume concept varied substantially between studies. Patient selection bias and publication bias are additional potential confounding factors. Consequently, prospective studies are required for standardization of the promising concept SBRT in the re-irradiation setting.

## Competing interests

The authors declare that they have no competing interests.

## Authors’ contributions

FM performed the literature review and drafted the manuscript; MG was involved in drafting the manuscript; MG and MF revised the manuscript critically for important intellectual content. All authors read and approved the final manuscript.

## Acknowledgements

This publication was funded by the German Research Foundation (DFG) and the University of Wuerzburg in the funding programme Open Access Publishing.

## References

[B1] Auperin A Le Pechoux C Rolland E Curran WJ Furuse K Fournel P Belderbos J Clamon G Ulutin HC Paulus R Meta-analysis of concomitant versus sequential radiochemotherapy in locally advanced non-small-cell lung cancer Journal of clinical oncology: official journal of the American Society of Clinical Oncology 2010 28 13 2181 2190 10.1200/JCO.2009.26.2543 20351327

[B2] Bourhis J Sire C Graff P Gregoire V Maingon P Calais G Gery B Martin L Alfonsi M Desprez P Concomitant chemoradiotherapy versus acceleration of radiotherapy with or without concomitant chemotherapy in locally advanced head and neck carcinoma (GORTEC 99–02): an open-label phase 3 randomised trial Lancet Oncol 2012 13 2 145 153 10.1016/S1470-2045(11)70346-1 22261362

[B3] Duenas-Gonzalez A Zarba JJ Patel F Alcedo JC Beslija S Casanova L Pattaranutaporn P Hameed S Blair JM Barraclough H Phase III, open-label, randomized study comparing concurrent gemcitabine plus cisplatin and radiation followed by adjuvant gemcitabine and cisplatin versus concurrent cisplatin and radiation in patients with stage IIB to IVA carcinoma of the cervix Journal of clinical oncology: official journal of the American Society of Clinical Oncology 2011 29 13 1678 1685 10.1200/JCO.2009.25.9663 21444871

[B4] Hofheinz RD Wenz F Post S Matzdorff A Laechelt S Hartmann JT Muller L Link H Moehler M Kettner E Chemoradiotherapy with capecitabine versus fluorouracil for locally advanced rectal cancer: a randomised, multicentre, non-inferiority, phase 3 trial Lancet Oncol 2012 13 6 579 588 10.1016/S1470-2045(12)70116-X 22503032

[B5] Stahl M Walz MK Stuschke M Lehmann N Meyer HJ Riera-Knorrenschild J Langer P Engenhart-Cabillic R Bitzer M Konigsrainer A Phase III comparison of preoperative chemotherapy compared with chemoradiotherapy in patients with locally advanced adenocarcinoma of the esophagogastric junction Journal of clinical oncology: official journal of the American Society of Clinical Oncology 2009 27 6 851 856 10.1200/JCO.2008.17.0506 19139439

[B6] Blackstock AW Govindan R Definitive chemoradiation for the treatment of locally advanced non small-cell lung cancer Journal of clinical oncology: official journal of the American Society of Clinical Oncology 2007 25 26 4146 4152 10.1200/JCO.2007.12.6581 17827465

[B7] Gressen EL Werner-Wasik M Cohn J Topham A Curran WJ Jr Thoracic reirradiation for symptomatic relief after prior radiotherapeutic management for lung cancer Am J Clin Oncol 2000 23 2 160 163 10.1097/00000421-200004000-00011 10776977

[B8] Chemoradiotherapy for Cervical Cancer Meta-analysis C Reducing uncertainties about the effects of chemoradiotherapy for cervical cancer: individual patient data meta-analysis Cochrane Database Syst Rev 2010 1 CD008285 10.1002/14651858.CD008285 20091664 PMC7105912

[B9] Chow E Harris K Fan G Tsao M Sze WM Palliative radiotherapy trials for bone metastases: a systematic review Journal of clinical oncology: official journal of the American Society of Clinical Oncology 2007 25 11 1423 1436 10.1200/JCO.2006.09.5281 17416863

[B10] Bentzen SM Constine LS Deasy JO Eisbruch A Jackson A Marks LB Ten Haken RK Yorke ED Quantitative analyses of normal tissue effects in the clinic (QUANTEC): an introduction to the scientific issues Int J Radiat Oncol Biol Phys 2010 76 3 Suppl S3 S9 20171515 10.1016/j.ijrobp.2009.09.040 PMC3431964

[B11] Medin PM Boike TP Spinal cord tolerance in the age of spinal radiosurgery: lessons from preclinical studies Int J Radiat Oncol Biol Phys 2011 79 5 1302 1309 10.1016/j.ijrobp.2010.10.052 21183290 PMC3074505

[B12] De Crevoisier R Bourhis J Domenge C Wibault P Koscielny S Lusinchi A Mamelle G Janot F Julieron M Leridant AM Full-dose reirradiation for unresectable head and neck carcinoma: experience at the Gustave-Roussy Institute in a series of 169 patients Journal of clinical oncology: official journal of the American Society of Clinical Oncology 1998 16 11 3556 3562 9817275 10.1200/JCO.1998.16.11.3556

[B13] Haque W Crane CH Krishnan S Delclos ME Javle M Garrett CR Wolff RA Das P Reirradiation to the abdomen for gastrointestinal malignancies Radiat Oncol 2009 4 55 10.1186/1748-717X-4-55 19922641 PMC2787526

[B14] Simmonds RH Hopewell JW Robbins ME Residual radiation-induced injury in dermal tissue: implications for retreatment Br J Radiol 1989 62 742 915 920 10.1259/0007-1285-62-742-915 2819360

[B15] Pomp J Levendag PC van Putten WL Reirradiation of recurrent tumors in the head and neck Am J Clin Oncol 1988 11 5 543 549 10.1097/00000421-198810000-00007 3177256

[B16] Stevens KR Jr Britsch A Moss WT High-dose reirradiation of head and neck cancer with curative intent Int J Radiat Oncol Biol Phys 1994 29 4 687 698 10.1016/0360-3016(94)90555-X 8040014

[B17] Salama JK Vokes EE Chmura SJ Milano MT Kao J Stenson KM Witt ME Haraf DJ Long-term outcome of concurrent chemotherapy and reirradiation for recurrent and second primary head-and-neck squamous cell carcinoma Int J Radiat Oncol Biol Phys 2006 64 2 382 391 10.1016/j.ijrobp.2005.07.005 16213104

[B18] Biagioli MC Harvey M Roman E Raez LE Wolfson AH Mutyala S Han HS Markoe A Intensity-modulated radiotherapy with concurrent chemotherapy for previously irradiated, recurrent head and neck cancer Int J Radiat Oncol Biol Phys 2007 69 4 1067 1073 10.1016/j.ijrobp.2007.04.057 17967302

[B19] Duprez F Madani I Bonte K Boterberg T Vakaet L Derie C De Gersem W De Neve W Intensity-modulated radiotherapy for recurrent and second primary head and neck cancer in previously irradiated territory Radiotherapy and oncology: journal of the European Society for Therapeutic Radiology and Oncology 2009 93 3 563 569 10.1016/j.radonc.2009.10.012 19919885

[B20] Terry NH Tucker SL Travis EL Residual radiation damage in murine lung assessed by pneumonitis Int J Radiat Oncol Biol Phys 1988 14 5 929 938 10.1016/0360-3016(88)90015-6 3360659

[B21] Ang KK Jiang GL Feng Y Stephens LC Tucker SL Price RE Extent and kinetics of recovery of occult spinal cord injury Int J Radiat Oncol Biol Phys 2001 50 4 1013 1020 10.1016/S0360-3016(01)01599-1 11429229

[B22] Medin PM Foster RD van der Kogel AJ Sayre JW McBride WH Solberg TD Spinal cord tolerance to reirradiation with single-fraction radiosurgery: a swine model Int J Radiat Oncol Biol Phys 2012 83 3 1031 1037 22197239 10.1016/j.ijrobp.2011.08.030 PMC3314093

[B23] Nieder C Grosu AL Andratschke NH Molls M Proposal of human spinal cord reirradiation dose based on collection of data from 40 patients Int J Radiat Oncol Biol Phys 2005 61 3 851 855 10.1016/j.ijrobp.2004.06.016 15708265

[B24] Sahgal A Ma L Gibbs I Gerszten PC Ryu S Soltys S Weinberg V Wong S Chang E Fowler J Spinal cord tolerance for stereotactic body radiotherapy Int J Radiat Oncol Biol Phys 2010 77 2 548 553 10.1016/j.ijrobp.2009.05.023 19765914

[B25] Gaze MN Kelly CG Kerr GR Cull A Cowie VJ Gregor A Howard GC Rodger A Pain relief and quality of life following radiotherapy for bone metastases: a randomised trial of two fractionation schedules Radiotherapy and oncology: journal of the European Society for Therapeutic Radiology and Oncology 1997 45 2 109 116 10.1016/S0167-8140(97)00101-1 9423999

[B26] Roos DE Turner SL O'Brien PC Smith JG Spry NA Burmeister BH Hoskin PJ Ball DL Trans-Tasman Radiation Oncology Group T Randomized trial of 8 Gy in 1 versus 20 Gy in 5 fractions of radiotherapy for neuropathic pain due to bone metastases (trans-tasman radiation oncology group, TROG 96.05) Radiotherapy and oncology: journal of the European Society for Therapeutic Radiology and Oncology 2005 75 1 54 63 10.1016/j.radonc.2004.09.017 15878101

[B27] Steenland E Leer JW van Houwelingen H Post WJ van den Hout WB Kievit J de Haes H Martijn H Oei B Vonk E The effect of a single fraction compared to multiple fractions on painful bone metastases: A global analysis of the dutch bone metastasis study Radiotherapy and oncology: journal of the European Society for Therapeutic Radiology and Oncology 1999 52 2 101 109 10.1016/S0167-8140(99)00110-3 10577695

[B28] van der Linden YM Lok JJ Steenland E Martijn H van Houwelingen H Marijnen CA Leer JW Dutch bone metastasis study G Single fraction radiotherapy is efficacious: A further analysis of the dutch bone metastasis study controlling for the influence of retreatment Int J Radiat Oncol Biol Phys 2004 59 2 528 537 10.1016/j.ijrobp.2003.10.006 15145173

[B29] Chang UK Cho WI Kim MS Cho CK Lee DH Rhee CH Local tumor control after retreatment of spinal metastasis using stereotactic body radiotherapy; comparison with initial treatment group Acta Oncol 2012 51 5 589 595 10.3109/0284186X.2012.666637 22414095

[B30] Choi CY Adler JR Gibbs IC Chang SD Jackson PS Minn AY Lieberson RE Soltys SG Stereotactic radiosurgery for treatment of spinal metastases recurring in close proximity to previously irradiated spinal cord Int J Radiat Oncol Biol Phys 2010 78 2 499 506 10.1016/j.ijrobp.2009.07.1727 20133079

[B31] Damast S Wright J Bilsky M Hsu M Zhang Z Lovelock M Cox B Zatcky J Yamada Y Impact of dose on local failure rates after image-guided reirradiation of recurrent paraspinal metastases Int J Radiat Oncol Biol Phys 2011 81 3 819 826 10.1016/j.ijrobp.2010.06.013 20888133

[B32] Garg AK Wang XS Shiu AS Allen P Yang J McAleer MF Azeem S Rhines LD Chang EL Prospective evaluation of spinal reirradiation by using stereotactic body radiation therapy: the university of texas MD anderson cancer center experience Cancer 2011 117 15 3509 3516 10.1002/cncr.25918 21319143

[B33] Mahadevan A Floyd S Wong E Jeyapalan S Groff M Kasper E Stereotactic body radiotherapy reirradiation for recurrent epidural spinal metastases Int J Radiat Oncol Biol Phys 2011 81 5 1500 1505 10.1016/j.ijrobp.2010.08.012 20950944

[B34] Mahan SL Ramsey CR Scaperoth DD Chase DJ Byrne TE Evaluation of image-guided helical tomotherapy for the retreatment of spinal metastasis Int J Radiat Oncol Biol Phys 2005 63 5 1576 1583 10.1016/j.ijrobp.2005.05.015 16125871

[B35] Milker-Zabel S Zabel A Thilmann C Schlegel W Wannenmacher M Debus J Clinical results of retreatment of vertebral bone metastases by stereotactic conformal radiotherapy and intensity-modulated radiotherapy Int J Radiat Oncol Biol Phys 2003 55 1 162 167 10.1016/S0360-3016(02)03864-6 12504049

[B36] Sahgal A Ames C Chou D Ma L Huang K Xu W Chin C Weinberg V Chuang C Weinstein P Stereotactic body radiotherapy is effective salvage therapy for patients with prior radiation of spinal metastases Int J Radiat Oncol Biol Phys 2009 74 3 723 731 10.1016/j.ijrobp.2008.09.020 19095374

[B37] Sterzing F Hauswald H Uhl M Herm H Wiener A Herfarth K Debus J Krempien R Spinal cord sparing reirradiation with helical tomotherapy Cancer 2010 116 16 3961 3968 10.1002/cncr.25187 20564110

[B38] Green N Melbye RW Lung cancer: retreatment of local recurrence after definitive irradiation Cancer 1982 49 5 865 868 10.1002/1097-0142(19820301)49:5<865::AID-CNCR2820490507>3.0.CO;2-H 7059924

[B39] Jackson MA Ball DL Palliative retreatment of locally-recurrent lung cancer after radical radiotherapy The Medical journal of Australia 1987 147 8 391 394 2443823 10.5694/j.1326-5377.1987.tb133559.x

[B40] Jeremic B Videtic GM Chest reirradiation with external beam radiotherapy for locally recurrent non-small-cell lung cancer: a review Int J Radiat Oncol Biol Phys 2011 80 4 969 977 10.1016/j.ijrobp.2011.01.069 21489714

[B41] Poltinnikov IM Fallon K Xiao Y Reiff JE Curran WJ Jr Werner-Wasik M Combination of longitudinal and circumferential three-dimensional esophageal dose distribution predicts acute esophagitis in hypofractionated reirradiation of patients with non-small-cell lung cancer treated in stereotactic body frame Int J Radiat Oncol Biol Phys 2005 62 3 652 658 10.1016/j.ijrobp.2004.10.030 15936541

[B42] Coon D Gokhale AS Burton SA Heron DE Ozhasoglu C Christie N Fractionated stereotactic body radiation therapy in the treatment of primary, recurrent, and metastatic lung tumors: the role of positron emission tomography/computed tomography-based treatment planning Clin Lung Cancer 2008 9 4 217 221 10.3816/CLC.2008.n.032 18650169

[B43] Kelly P Balter PA Rebueno N Sharp HJ Liao Z Komaki R Chang JY Stereotactic body radiation therapy for patients with lung cancer previously treated with thoracic radiation Int J Radiat Oncol Biol Phys 2010 78 5 1387 1393 10.1016/j.ijrobp.2009.09.070 20381271 PMC3401019

[B44] Peulen H Karlsson K Lindberg K Tullgren O Baumann P Lax I Lewensohn R Wersall P Toxicity after reirradiation of pulmonary tumours with stereotactic body radiotherapy Radiotherapy and oncology: journal of the European Society for Therapeutic Radiology and Oncology 2011 101 2 260 266 10.1016/j.radonc.2011.09.012 22056534

[B45] McLaughlin MP Parsons JT Fein DA Stringer SP Cassisi NJ Mendenhall WM Million RR Salvage surgery after radiotherapy failure in T1-T2 squamous cell carcinoma of the glottic larynx Head Neck 1996 18 3 229 235 10.1002/(SICI)1097-0347(199605/06)18:3<229::AID-HED4>3.0.CO;2-1 8860763

[B46] Wong LY Wei WI Lam LK Yuen AP Salvage of recurrent head and neck squamous cell carcinoma after primary curative surgery Head Neck 2003 25 11 953 959 10.1002/hed.10310 14603456

[B47] Forastiere AA Metch B Schuller DE Ensley JF Hutchins LF Triozzi P Kish JA McClure S VonFeldt E Williamson SK Randomized comparison of cisplatin plus fluorouracil and carboplatin plus fluorouracil versus methotrexate in advanced squamous-cell carcinoma of the head and neck: a Southwest Oncology Group study Journal of clinical oncology: official journal of the American Society of Clinical Oncology 1992 10 8 1245 1251 1634913 10.1200/JCO.1992.10.8.1245

[B48] Schrijvers D Johnson J Jiminez U Gore M Kosmidis P Szpirglas H Robbins K Oliveira J Lewensohn R Schuller J Phase III trial of modulation of cisplatin/fluorouracil chemotherapy by interferon alfa-2b in patients with recurrent or metastatic head and neck cancer Head and Neck Interferon Cooperative Study Group. Journal of clinical oncology: official journal of the American Society of Clinical Oncology 1998 16 3 1054 1059 10.1200/JCO.1998.16.3.1054 9508190

[B49] Voynov G Heron DE Burton S Grandis J Quinn A Ferris R Ozhasoglu C Vogel W Johnson J Frameless stereotactic radiosurgery for recurrent head and neck carcinoma Technol Cancer Res Treat 2006 5 5 529 535 16981796 10.1177/153303460600500510

[B50] Roh KW Jang JS Kim MS Sun DI Kim BS Jung SL Kang JH Yoo EJ Yoon SC Jang HS Fractionated stereotactic radiotherapy as reirradiation for locally recurrent head and neck cancer Int J Radiat Oncol Biol Phys 2009 74 5 1348 1355 10.1016/j.ijrobp.2008.10.013 19117695

[B51] Unger KR Lominska CE Deeken JF Davidson BJ Newkirk KA Gagnon GJ Hwang J Slack RS Noone AM Harter KW Fractionated stereotactic radiosurgery for reirradiation of head-and-neck cancer Int J Radiat Oncol Biol Phys 2010 77 5 1411 1419 10.1016/j.ijrobp.2009.06.070 20056341

[B52] Heron DE Ferris RL Karamouzis M Andrade RS Deeb EL Burton S Gooding WE Branstetter BF Mountz JM Johnson JT Stereotactic body radiotherapy for recurrent squamous cell carcinoma of the head and neck: results of a phase I dose-escalation trial Int J Radiat Oncol Biol Phys 2009 75 5 1493 1500 10.1016/j.ijrobp.2008.12.075 19464819

[B53] Heron DE Rwigema JC Gibson MK Burton SA Quinn AE Ferris RL Concurrent cetuximab with stereotactic body radiotherapy for recurrent squamous cell carcinoma of the head and neck: a single institution matched case–control study Am J Clin Oncol 2011 34 2 165 172 20686406 10.1097/COC.0b013e3181dbb73e

[B54] Comet B Kramar A Faivre-Pierret M Dewas S Coche-Dequeant B Degardin M Lefebvre JL Lacornerie T Lartigau EF Salvage stereotactic reirradiation with or without cetuximab for locally recurrent head-and-neck cancer: a feasibility study Int J Radiat Oncol Biol Phys 2012 84 1 203 209 10.1016/j.ijrobp.2011.11.054 22331006

[B55] Ozyigit G Cengiz M Yazici G Yildiz F Gurkaynak M Zorlu F Yildiz D Hosal S Gullu I Akyol F A retrospective comparison of robotic stereotactic body radiotherapy and three-dimensional conformal radiotherapy for the reirradiation of locally recurrent nasopharyngeal carcinoma Int J Radiat Oncol Biol Phys 2011 81 4 e263 e268 10.1016/j.ijrobp.2011.02.054 21514737

[B56] Chang JT See LC Liao CT Ng SH Wang CH Chen IH Tsang NM Tseng CK Tang SG Hong JH Locally recurrent nasopharyngeal carcinoma Radiotherapy and oncology: journal of the European Society for Therapeutic Radiology and Oncology 2000 54 2 135 142 10.1016/S0167-8140(99)00177-2 10699476

[B57] Chua DT Wu SX Lee V Tsang J Comparison of single versus fractionated dose of stereotactic radiotherapy for salvaging local failures of nasopharyngeal carcinoma: a matched-cohort analysis Head Neck Oncol 2009 1 13 10.1186/1758-3284-1-13 19463191 PMC2694191

[B58] Lee AW Law SC Foo W Poon YF Cheung FK Chan DK Tung SY Thaw M Ho JH Retrospective analysis of patients with nasopharyngeal carcinoma treated during 1976–1985: survival after local recurrence Int J Radiat Oncol Biol Phys 1993 26 5 773 782 10.1016/0360-3016(93)90491-D 8344845

[B59] Pai PC Chuang CC Wei KC Tsang NM Tseng CK Chang CN Stereotactic radiosurgery for locally recurrent nasopharyngeal carcinoma Head Neck 2002 24 8 748 753 10.1002/hed.10116 12203799

[B60] Vargo JA Wegner RE Heron DE Ferris RL Rwigema JC Quinn A Gigliotti P Ohr J Kubicek GJ Burton S Stereotactic body radiation therapy for locally recurrent, previously irradiated nonsquamous cell cancers of the head and neck Head Neck 2012 34 8 1153 1161 10.1002/hed.21889 22076812

[B61] Heron JT Trassin M Ashraf K Gajek M He Q Yang SY Nikonov DE Chu YH Salahuddin S Ramesh R Electric-field-induced magnetization reversal in a ferromagnet-multiferroic heterostructure Phys Rev Lett 2011 107 21 217202 22181917 10.1103/PhysRevLett.107.217202

[B62] Heron J Hickman M Macleod J Munafo MR Characterizing patterns of smoking initiation in adolescence: comparison of methods for dealing with missing data Nicotine & tobacco research: official journal of the Society for Research on Nicotine and Tobacco 2011 13 12 1266 1275 10.1093/ntr/ntr161 PMC3223580 21994336

[B63] Dewas S Bibault JE Mirabel X Nickers P Castelain B Lacornerie T Jarraya H Lartigau E Robotic image-guided reirradiation of lateral pelvic recurrences: preliminary results Radiat Oncol 2011 6 77 10.1186/1748-717X-6-77 21699690 PMC3141526

[B64] Guckenberger M Bachmann J Wulf J Mueller G Krieger T Baier K Richter A Wilbert J Flentje M Stereotactic body radiotherapy for local boost irradiation in unfavourable locally recurrent gynaecological cancer Radiotherapy and oncology: journal of the European Society for Therapeutic Radiology and Oncology 2010 94 1 53 59 10.1016/j.radonc.2009.12.004 20079550

[B65] Deodato F Macchia G Grimaldi L Ferrandina G Lorusso D Salutari V Cilla S Valentini V Cellini N Piermattei A Stereotactic radiotherapy in recurrent gynecological cancer: a case series Oncol Rep 2009 22 2 415 419 19578785

[B66] Jereczek-Fossa BA Beltramo G Fariselli L Fodor C Santoro L Vavassori A Zerini D Gherardi F Ascione C Bossi-Zanetti I Robotic image-guided stereotactic radiotherapy, for isolated recurrent primary, lymph node or metastatic prostate cancer Int J Radiat Oncol Biol Phys 2012 82 2 889 897 10.1016/j.ijrobp.2010.11.031 21277113

[B67] Defoe SG Bernard ME Rwigema JC Heron DE Ozhasoglu C Burton S Stereotactic body radiotherapy for the treatment of presacral recurrences from rectal cancers J Cancer Res Ther 2011 7 4 408 411 10.4103/0973-1482.92000 22269400

[B68] Pectasides D Kamposioras K Papaxoinis G Pectasides E Chemotherapy for recurrent cervical cancer Cancer Treat Rev 2008 34 7 603 613 10.1016/j.ctrv.2008.05.006 18657909

[B69] Unger ZM Gonzalez JL Hanissian PD Schned AR Pseudolipomatosis in hysteroscopically resected tissues from the gynecologic tract: pathologic description and frequency Am J Surg Pathol 2009 33 8 1187 1190 10.1097/PAS.0b013e3181a0d3a6 19440147

[B70] Heron M Van Moorsel CH Grutters JC Huizinga TW Van der Helm-van Mil AH Nagtegaal MM Ruven HJ Van den Bosch JM Genetic variation in GREM1 is a risk factor for fibrosis in pulmonary sarcoidosis Tissue Antigens 2011 77 2 112 117 10.1111/j.1399-0039.2010.01590.x 21214523

